# High Performance Flip-Structure Enhancement-Mode HEMT with Face-to-Face Double Gates

**DOI:** 10.1186/s11671-022-03713-4

**Published:** 2022-08-11

**Authors:** Siyu Deng, Jie Wei, Cheng Zhang, Dezun Liao, Tao Sun, Kemeng Yang, Lufan Xi, Bo Zhang, Xiaorong Luo

**Affiliations:** The State Key Laboratory of Electronic Thin Films and Integrated Devices, University of Electronic Science and Technology of China, Chengdu, 610054 China

**Keywords:** AlGaN/GaN HEMT, Enhancement-mode, Double gates, Flip-structure, Breakdown voltage

## Abstract

A novel double gates flip-structure enhancement-mode (E-mode) high electron mobility transistor with step field plate (DFF HEMT) is proposed. It features face-to-face double gates, including a top trench MIS gate with a step field plate and a bottom planar MIS gate, which is shorted together. In the on-state, the double gates not only can restore the 2DEG by the higher electric potential, but also can form the electron accumulation layers, and thus increase the saturation output current and reduce the on-resistance. The face-to-face double gates together deplete the 2DEG by using the work function difference to realize E-mode, instead of by etching the AlGaN layer under the gate for the conventional MIS gate E-mode HEMT. The double-gate structure not only avoids etch damage, but also maintains both high threshold voltage and low on-resistance. Meanwhile, the step gate field plate modulates *E*-field distribution to increase the *BV*. In order to easily fabricate, the trench gate with step field plate must be located on the top of device, forming the flip-structure. The flip-structure is also beneficial to decrease the leakage current in the substrate. The simulated *V*_th_, *BV* and *I*_d_ of the DFF HEMT are 0.8 V, 465 V and 494 mA/mm, respectively. The *FOM* of the DFF HEMT is 79.8% and 444.2% higher than those of the conventional MIS*-*FP HEMT and MIS HEMT.

## Introduction

GaN-based high electron mobility transistor (HEMT) has great prospects in low loss and high power application [1–4], owing to its high density two-dimensional electron gas (2DEG) and high electron mobility. The conventional GaN HEMT is always depletion mode (D-mode), while the enhancement mode (E-mode) is necessary in power electronics applications. Many structures have been proposed to achieve the E-mode, such as a thinned AlGaN barrier layer [5], p-gate structure [6], recessed gate structure [7], and fluoride ion treatment [8, 9]. All technologies above are realized by depleting the 2DEG under the gate, inevitably encountering a tradeoff between a high threshold voltage (*V*_th_) and a large saturated output current (*I*_d, sat_). Meanwhile, the gate Metal-gate Insulator-AlGaN Semiconductor (MIS) generally has been used for the HEMT with recessed gate.

The breakdown voltage (*BV)* of the GaN HEMT is far below its theoretical limit because the E-field crowding causes premature breakdown at the edge of gate. To solve this problem, several technologies have been adopted, including field plate (FP) [10, 11], RESURF technology [12, 13] and polarization junction concept [14, 15]. However, there is also a tradeoff relationship between the high *BV* and *I*_d, sat_, owning to the assisted depletion on the 2DEG of the drift region.

Recently, a flip-structure has been proposed [16]. By the substrate transferring technology, the device is located on the poly-AlN, which reduces the leakage of Si substrate. Importantly, the flip-structure facilitates the monolithic integration of the HEMT and the vertical LED.

In this paper, a novel double gates flip-structure enhancement-mode (E-mode) HEMT with field plate (DFF-HEMT) is proposed. Its on-state and off-state electrical characteristics are studied by simulation. The simulated results show that DFF-HEMT can significantly improve the on-state, off-state characteristics and realize E-mode.

## Device Structure and Mechanism

**Fig. 1:**
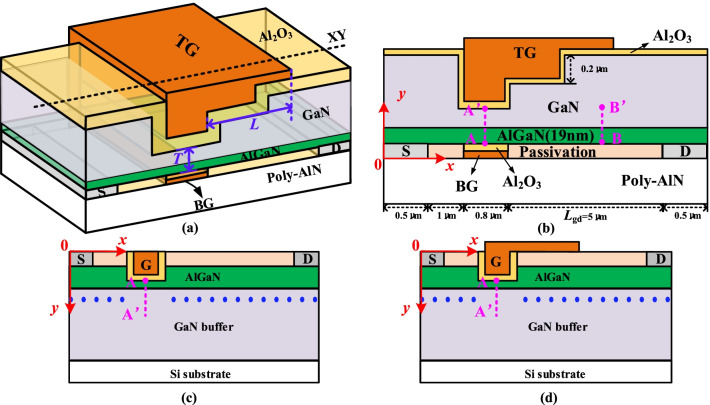
**a** Schematic section of the DFF HEMT and schematic cross section of the **b** DFF HEMT (at XY), **c** MIS HEMT and **d** MIS-FP HEMT

Figure 1a and b are the schematic section of the proposed DFF HEMT. The DFF HEMT has the following two features. One is the face-to-face double gates, including a top trench MIS gate with step field plate (TG) and a bottom planar MIS gate (BG), and they are shorted together, as shown in Fig. 1a. In order to easily fabricate, the trench gate with step field plate must be located on the top of device. So, the other feature is a flip-structure, of which the source, the bottom gate and the drain are below the 2DEG channel instead of above the 2DEG channel. The length of the step field plate is *L*, and the thickness of the semiconductor between the double gates is *T* as shown in Fig. 1b, and *T *= 40 nm and *L* = 2.6 μm are fixed except especial statement. The thickness of Al_2_O_3_ layer is 10 nm and the height of the step field plate is 200 nm. The *x-* and *y-*direction are given. The conventional MIS trench gate HEMT (MIS HEMT) and the MIS trench gate HEMT with field plate (MIS-FP HEMT) are shown in Fig. 1c and d. The thickness of passivation layer, AlGaN barrier layer and GaN buffer layer are 50 nm, 19 nm and 1 μm for three devices, and the lateral dimensions of drift region *L*_gd_ = 5 μm (the distance between the gate and drain) for the two device is the same as that of the DFF HEMT. The length of the field plate is 2.3 μm for the MIS-FP HEMT. The metal work function is 5.15 eV. Several important physical models have also been considered, such as narrowing of the band gap, high field saturation, doping dependency and Shockley–Read–Hall [17]. The mobility of the 2DEG under the gate has been set as 680 cm^2^/V·s [18] and the electron mobility of the MIS interface has been set as 100 cm^2^/V·s [19] because of the etch damage.

**Fig. 2:**
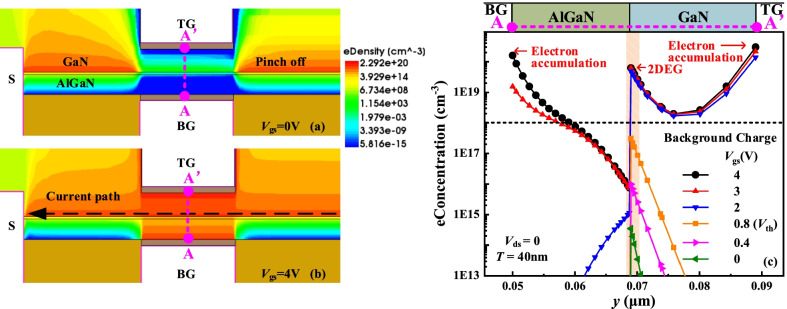
Electron concentration contours at **a** *V*_gs_ = 0 V, **b** *V*_gs_ = 4 V and **c** electron concentration distributions with different *V*_gs_ along AA’ line

The gate metal, Al_2_O_3_ and semiconductor AlGaN or GaN constitute top and bottom two MIS structures. Figure 2 shows the electron concentration contours and distributions along AA’ line under the different *V*_gs_ values. At *V*_gs_ = 0, 2DEG between the double gates is almost depleted by the double gates owing to the work function difference between the gate metal and AlGaN or GaN, as shown in Fig. 2a and c. Therefore, the current path is pinched off and E-mode is realized. The conventional MIS gate realizes the E-mode by etching the AlGaN layer to reduce 2DEG density, which not only introduces etch damage, but also increases the on-resistance and weakens the current capacity. For the DFF HEMT at *V*_gs_ > *V*_th_, the 2DEG completely recovers and even form an electron accumulation layer, as shown in Fig. 2b and c. The current path is turned on and effectively increases the output current. As *V*_gs_ increases, the 2DEG concentration increases in Fig. 2c. Furthermore, the electron concentration near the sides of two gates is higher than the doping concentration, verifying forming the electron accumulation layer. It breaks through the tradeoff relationship between the high *V*_th_ and the high output current in GaN HEMT. In the off state, the step field plate effectively improves the *BV* of the DFF HEMT.

## Results and Discussion

**Fig. 3:**
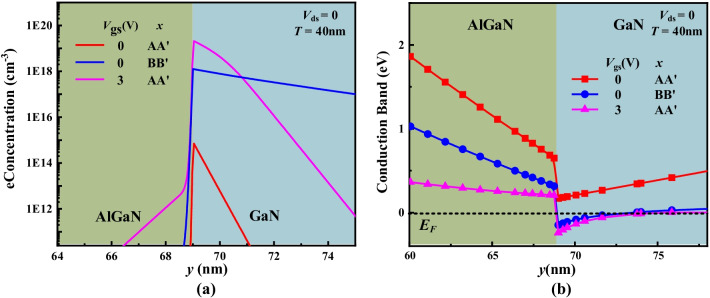
**a** Electron concentration distributions and **b** conduction band of DFF HEMT at *V*_gs_ = 0 V and *V*_gs_ = 3 V

Figure 3 shows the electron concentration distributions and conduction band of the DFF HEMT. When *V*_gs_ = 0, the electron density along AA’ line (in Fig. 1) is just 5 × 10^14^ cm^−3^ in Fig. 3a, and the conduction band is above the Fermi level in Fig. 3b. The E-mode is thus realized. When *V*_gs_ = 3 V > *V*_th_, the conduction band along AA’ line has been pulled below the Fermi level, and electron density is as high as 2 × 10^19^ cm^−3^. The DFF HEMT has been turned on. Additional, the electron density along AA’ line at *V*_gs_ = 3 V is higher than that along BB’ line at *V*_gs_ = 0 V in Fig. 3a. It indicates that 2DEG has been restored owing to high positive-voltage biased to the double gate.

**Fig. 4:**
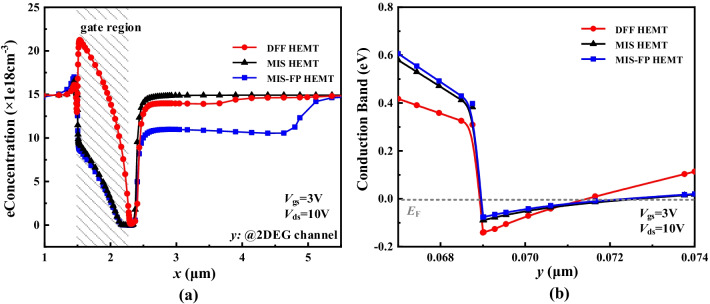
**a** 2DEG distributions and **b** conduction band along AA’ line for three devices in the on-state

Figure 4a shows the electron concentration distributions along the 2DEG channel in the on-state. The 2DEG concentration of the gate region of the DFF HEMT is much higher than those of the MIS HEMT and the MIS-FP HEMT. On one hand, the potential in the gate region of the DFF HEMT is much higher because both the BG and TG are applied to 3 V. The conduction band of the DFF HEMT is thus much lower as shown in Fig. 4b, which contributes to the higher 2DEG concentration. On the other hand, the polarization effect under the gate of the MIS HEMT and the MIS-FP HEMT is weakened because the AlGaN layer is etched partly to realize E-mode. Additional, it is normal that the 2DEG density under the right side of the gate is low for the three devices, because there is a higher electric potential of the semiconductor than that of the gate, as a depletion region. The field plate of the DFF HEMT is far away from the 2DEG channel and has smaller depletion effect on the 2DEG concentration than that of the MIS-FP HEMT. Therefore, the 2DEG concentration under the field plate of the DFF HEMT is higher, which contributes to higher current and lower specific on-resistance (*R*_on, sp_).

**Fig. 5:**
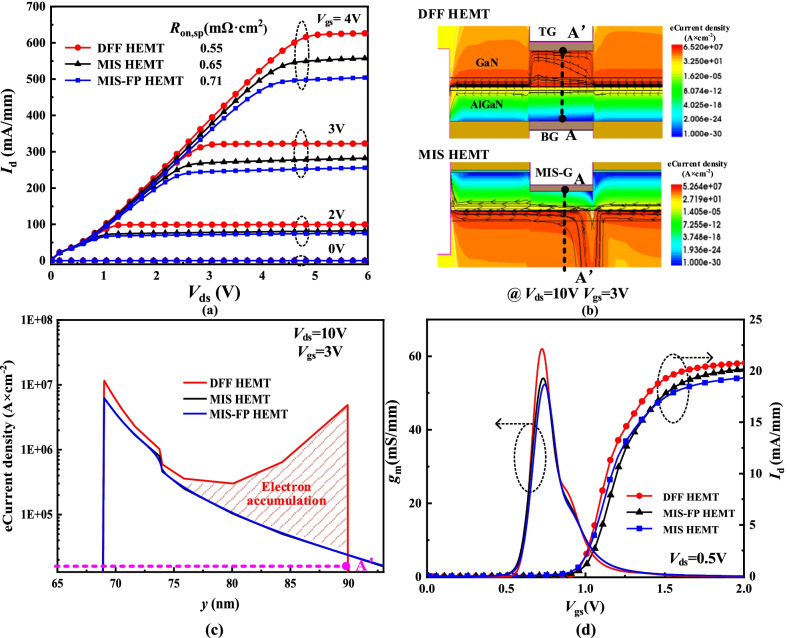
**a** output *I-V* characteristics, **b** electron current density, **c** electron current density distributions along AA’ line and **d** transfer characteristics for three devices

Figure 5 shows the output characteristic and transfer characteristic of the three devices. It can be seen from Fig. 5a that the DFF HEMT has the largest *I*_d, sat_ and the smallest *R*_on, sp_. The *I*_d, sat_ of the MIS-FP HEMT is the smallest because the field plate assists in depleting 2DEG along the drift region (see Fig. 4a). For the DFF HEMT, on one hand, the 2DEG is recovered, and on the other hand, the double gates introduce electron accumulation layer. Figure 5c shows the electron current density along AA’ line. The DFF HEMT has a large current density in electron accumulation layer, in addition to the 2DEG channel current. The 2DEG channel still plays a dominant role in the transport. It can be seen from Fig. 5d that DFF HEMT has the highest transconductance owing to the double gates. The *V*_th_ of three devices is designed as 0.8 V.

**Fig. 6:**
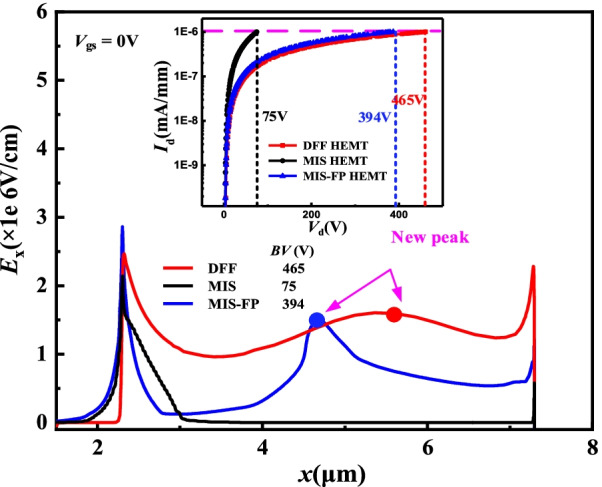
*E*_x_ distributions and *I-V* curves for three devices at breakdown at *y* = 0.051 μm

Figure 6 shows the lateral component of the *E*-field (*E*_*x*_) distribution and *I*-*V* curves at breakdown. It indicates that the *E*_*x*_ for the DFF HEMT is effectively improved. Compared with the MIS HEMT, the field plate not only brings out new *E*-field peak, but also expands the depletion region for the MIS-FP and DFF HEMT. Particularly, for the DFF HEMT, the step field plate further uniforms the *E*-field distribution in the drift region. Meanwhile, the leakage current of the substrate has been suppressed by the poly-AlN substrate layer. Therefore, the DFF HEMT achieves the highest *BV* of 465* V* (at *I*_d_ = 10^–6^ mA/mm), and *BV* is 394 V for the MIS-FP HEMT, as shown in Fig. 6. Without the field plate and flip-structure for the MIS HEMT, the drift region cannot be completely depleted and leakage current is large, and thus the *BV* is just 75 V at the same drift region length.

**Fig. 7:**
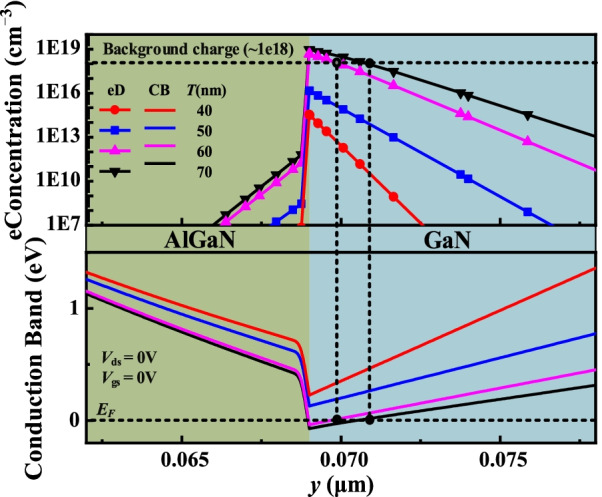
Electron concentration distributions and conduction band at *V*_gs_ = 0 V along AA’ line with the different *T* values

**Fig. 8:**
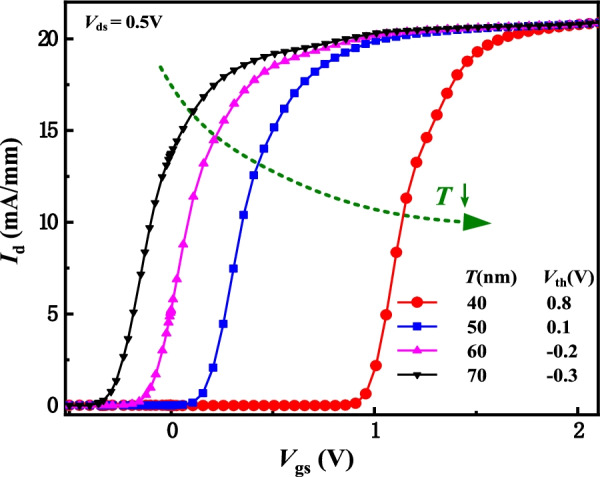
Transfer characteristics with the different *T* values

Figure 7 shows the electron concentration distributions and conduction band at *V*_gs_ = 0 V along AA' line with different *T* values. As *T* decreases, the conduction band rises and the electron density decreases. When *T* ≤ 50 nm, the depletion effect of the MIS structure on electrons is enhanced and the polarization effect is weakened. The whole conduction band is raised above *E*_*F*_. Consequently, E-mode is realized. When *T* > 50 nm, the depletion effect is weakened and the polarization effect is enhanced. Therefore, potential well of electrons is formed and the electron concentration rises to 10^19^ cm^−3^ order of magnitude, which is higher than the background charge concentration of 10^18^ cm^−3^. Figure 8 shows the transfer characteristic with different *T* values. The *V*_th_ increases as *T* decreases.

**Fig. 9:**
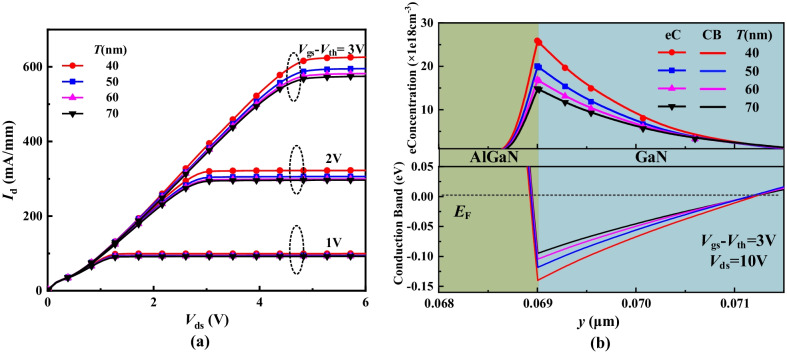
**a** output characteristics and **b** electron concentration distributions and conduction band (*V*_gs _− *V*_th_ = 3 V and *V*_ds_ = 10 V along AA’ line) with the different* T* values

Figure 9 shows the output characteristic, electron concentration distributions and conduction band with the different *T* values at a constant of (*V*_gs _− *V*_th_). The *I*_d, sat_ increases and the *R*_on, sp_ decrease as *T* decreases because the electron concentration increases, as shown in Fig. 9a and b. As *T* decreases, the *V*_th_ increases (in Fig. 8) and thus *V*_gs_ accordingly increases to maintain a constant of (*V*_gs _− *V*_th_), leading to the increase in electron concentration. Meanwhile, the conduction band is lower and the 2DEG concentration is higher owing to the lower *T* and higher *V*_gs_, as indicated in Fig. 9b. Different from the conventional GaN HEMT, the DFF HEMT break through the tradeoff relationship between the high *V*_th_ and high *I*_d, sat_. As *T* decreases, both the *I*_d,sat_ and the *V*_th_ increase.

**Fig. 10:**
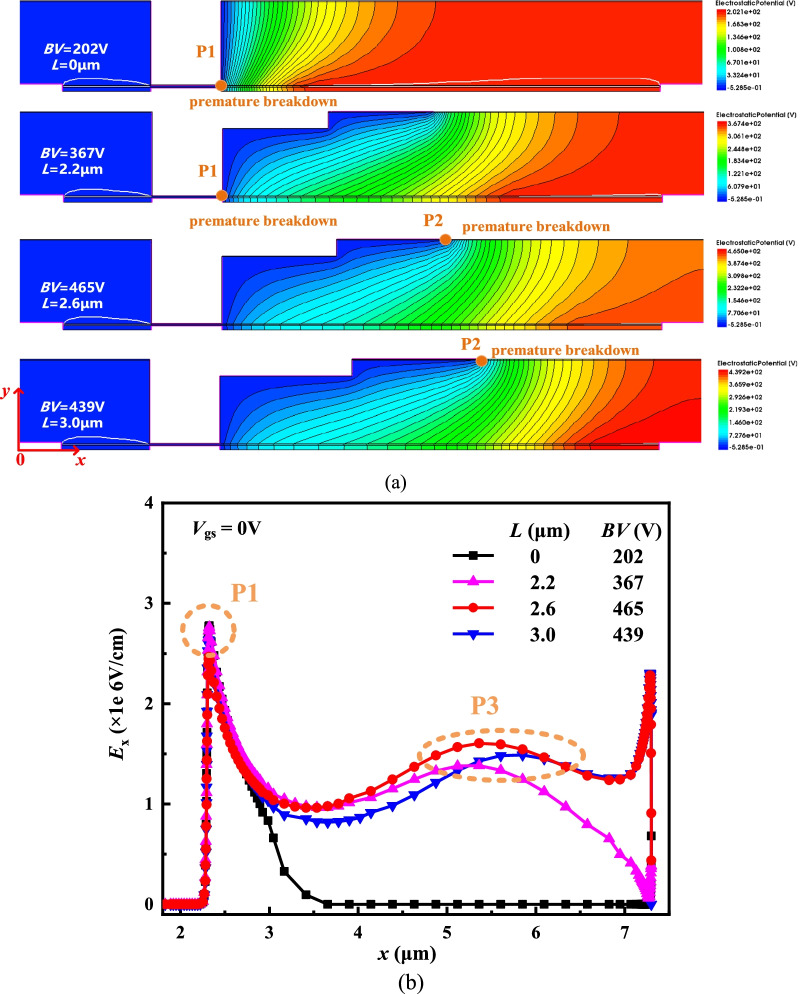
**a** Equipotential contours and **b** *Ex* distribution at breakdown at *y* = 0.051 μm with different *L* values

Figure 10 shows the influence of the *L* on the potential contours and *E*_*x*_ distribution of the DFF HEMT. The *BV* of the DFF HEMT firstly increases and then decreases as the *L* increases. When *L* = 0 μm means the DFF HEMT without the field plate, the *BV* is lower because the drift region has not been depleted. With the increase in *L* at *L* < 2.6 μm, the depletion region expands. The step field plate decreases the E-field peak at the gate edge (P1) and induces a new E-field peak below the end of the field plate (P2), so the *BV* increases as shown in Fig. 10b. P3 is the new E-field peak caused by the step field plate at *y* = 0.051 μm in Fig. 10b. At this time, the premature breakdown occurs at the right edge of the gate. When *L* = 2.6 μm, the *E*_*x*_ of the DFF HEMT is almost uniform and the *BV* of 465 V achieves the highest. When *L* = 3.0 μm, premature breakdown occurs owing to high P2, and *BV* decreases.

**Fig. 11:**
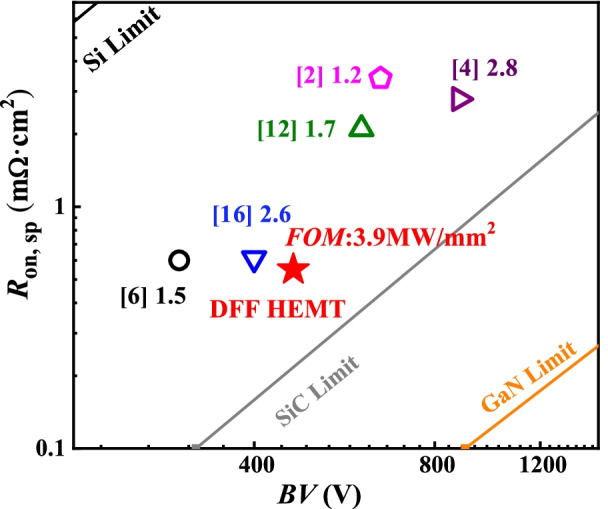
*R*_on,sp_ and off-state *BV* in the DFF-HEMT and reported AlGaN/GaN HEMTs, together with the theoretical Si-, SiC- and GaN- limit

Figure 11 demonstrates the *R*_on,sp_ and *BV* values of the DFF HEMT and reported AlGaN/GaN HEMTs, the DFF-HEMT has a higher figure of merit (FOM = *BV*^2^/*R*_on,sp_) than those of the AlGaN/GaN HEMTs in studies. As a result, when *T* = 40 nm, *L* = 2.6 μm, an excellent tradeoff between the on-state characteristics and the off-state characteristics is achieved.

**Fig. 12:**
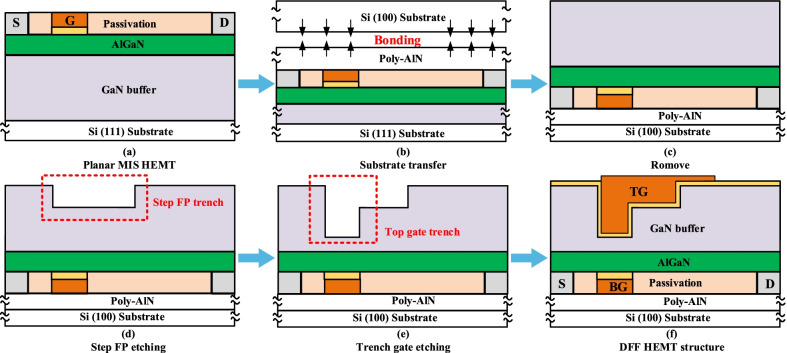
Key fabrication process flows for the DFF HEMT

The fabrication process steps of the DFF HEMT are shown in Fig. 12, which is referred to the experiment in [16]. Key processes are given as follows: (a) Fabricate a conventional MIS HEMT on a Si (111)-based AlGaN/GaN heterojunction wafer. (b) form poly-AlN film on the device surface by physical vapor deposition (PVD), and bonding to the Si (100) wafer. (c) Remove the Si (111) substrate and part of the GaN epitaxial layer. (d) form the shallow trench of the field plate by inductively coupled plasma (ICP) etching. (e) ICP etch TG deep trench. (f) implement Al_2_O_3_ layer and the top gate metal. Compared with those of Ref. [16], the fabrication process of the DFF HEMT only add twice ICP etchings ((d) and (e)) to realize the E-mode and further improve the *BV*. The DFF HEMT simultaneously realizes E-mode, and achieves a higher *BV* and a smaller *R*_on_.

## Conclusion

A novel double-gate flip-device enhancement-mode (E-mode) HEMT is proposed and investigated. It features face-to-face double gates with a top gate with step field plate and a bottom gate. The double gates restore the 2DEG and form the accumulation layers to increase the output current capacity and reduce the on-resistance. The double MIS gates together deplete the 2DEG to realize E-mode by the work function difference. Meanwhile, the step field plate flattens the *E*-field distribution and increase the *BV*. The proposed E-mode GaN power device has many advantages, including E-mode, high voltage and high saturation current capacity. The *V*_th_, *BV* and *I*_d_ of the DFF HEMT are 0.8 V, 465 V and 494 mA/mm, respectively. The *FOM* of the DFF HEMT is 79.8% and 444.2% higher than that of the conventional MIS-FP HEMT and MIS HEMT.

## Data Availability

The data used and analyzed during the current study are available from the corresponding authors upon reasonable request.

## Abbreviations

HEMT: High electron mobility transistor
E-mode: Enhancement mode
D-mode: Depletion mode
2DEG: Two-dimensional electron gas
MIS: Metal–insulator–semiconductor
FP: Field plate
DFF-HEMT: Double gates flip-structure E-mode HEMT with FP
MIS-FP HEMT: The MIS trench gate HEMT with FP
TG: Top gate
BG: Bottom gate
